# Supplementary material for: Visible-Light-Driven Degradation of Biological Contaminants on the Surface of Textile Fabric Modified with TiO2-N Photocatalyst
Source: Int J Mol Sci. 2025 Aug 5;26(15):7550. doi: 10.3390/ijms26157550 (PMC12347451; doi:10.3390/ijms26157550)
Supplement: Supplementary file 1 [file ijms-26-07550-s001.zip › ijms-3759979-supplementary.pdf]

Supporting information
for

Visible-light-driven degradation of biological contaminants on the surface of textile fabric modified with TiO_2-N photocatalyst

by

Maria Solovyeva [1,2], Evgenii Zhuravlev [3], Yuliya Kozlova [3], Alevtina Bardasheva [3], Vera Morozova [3], Grigory Stepanov [3], Denis Kozlov [1,2], Mikhail Lyulyukin [1,2], Dmitry Selishchev [1,2,*]

[1]Research and Educational Center "Institute of Chemical Technologies", Novosibirsk State University, Pirogova St. 2, Novosibirsk 630090, Russia;
[2]Competence Center of the National Technological Initiative "Hydrogen as the Basis of a Low-Carbon Economy", Lavrentieva Ave. 7, Novosibirsk 630090, Russia;
[3]Institute of Chemical Biology and Fundamental Medicine SB RAS, Lavrentieva Ave. 8, Novosibirsk 630090, Russia
* Correspondence: d.selishchev@g.nsu.ru

1. XPS spectra of TN-Cu, PF-TN, and PF-TN-Cu samples in (a) N 1s and (b) Cu 2p regions

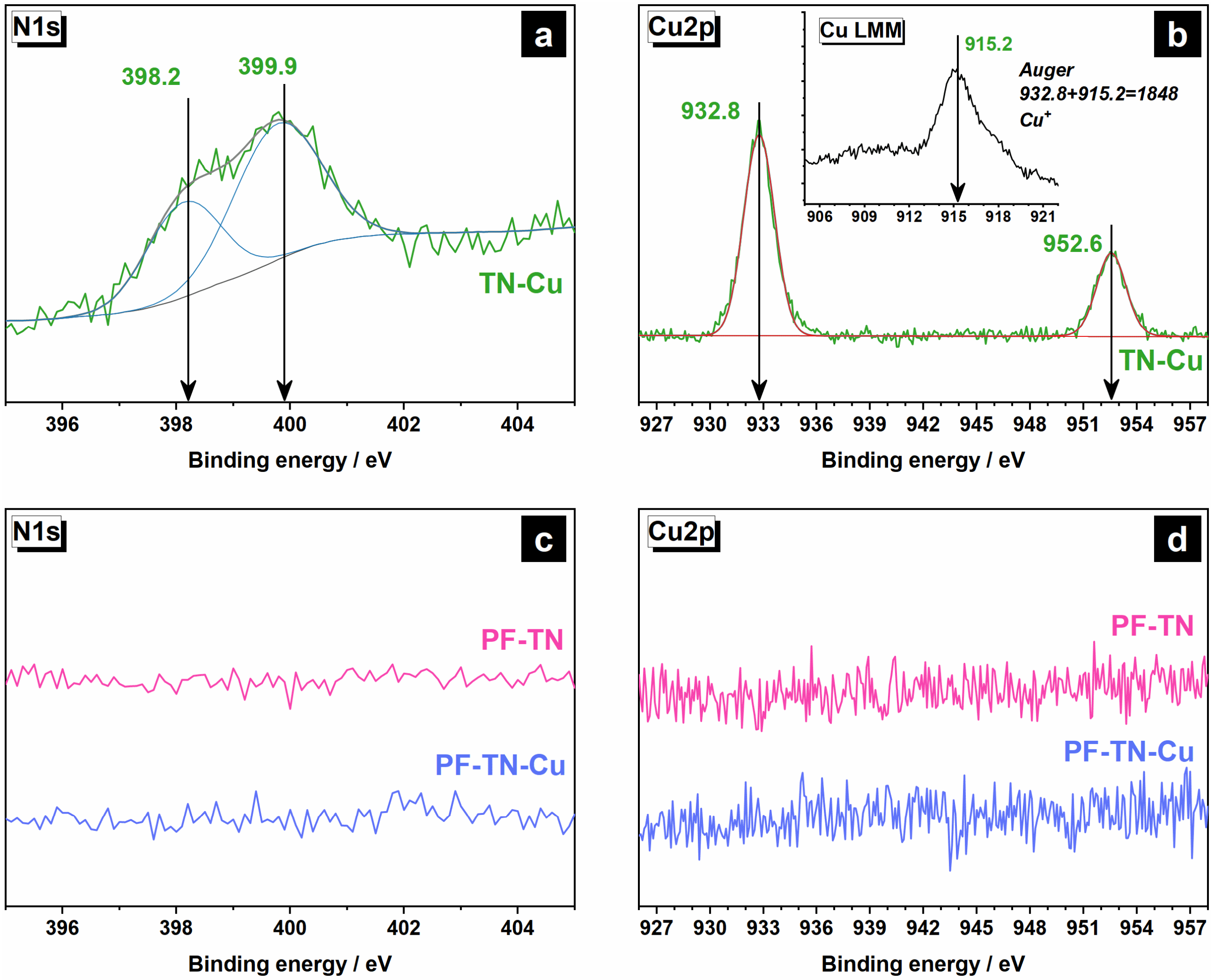

Figure S1. XPS spectra of Cu-modified TiO_2-N (**TN-Cu**) powder with Cu loading of 1 wt.% in (a) N 1s and (b) Cu 2p spectral regions; XPS spectra of photoactive fabric modified with TiO_2-N (**PF-TN)** and Cu-modified photoactive fabric with TiO_2-N (**PF-TN-Cu**) in (c) N 1s and (d) Cu 2p spectral regions.

2. Photocatalytic activity of powdered TN-Cu photocatalyst

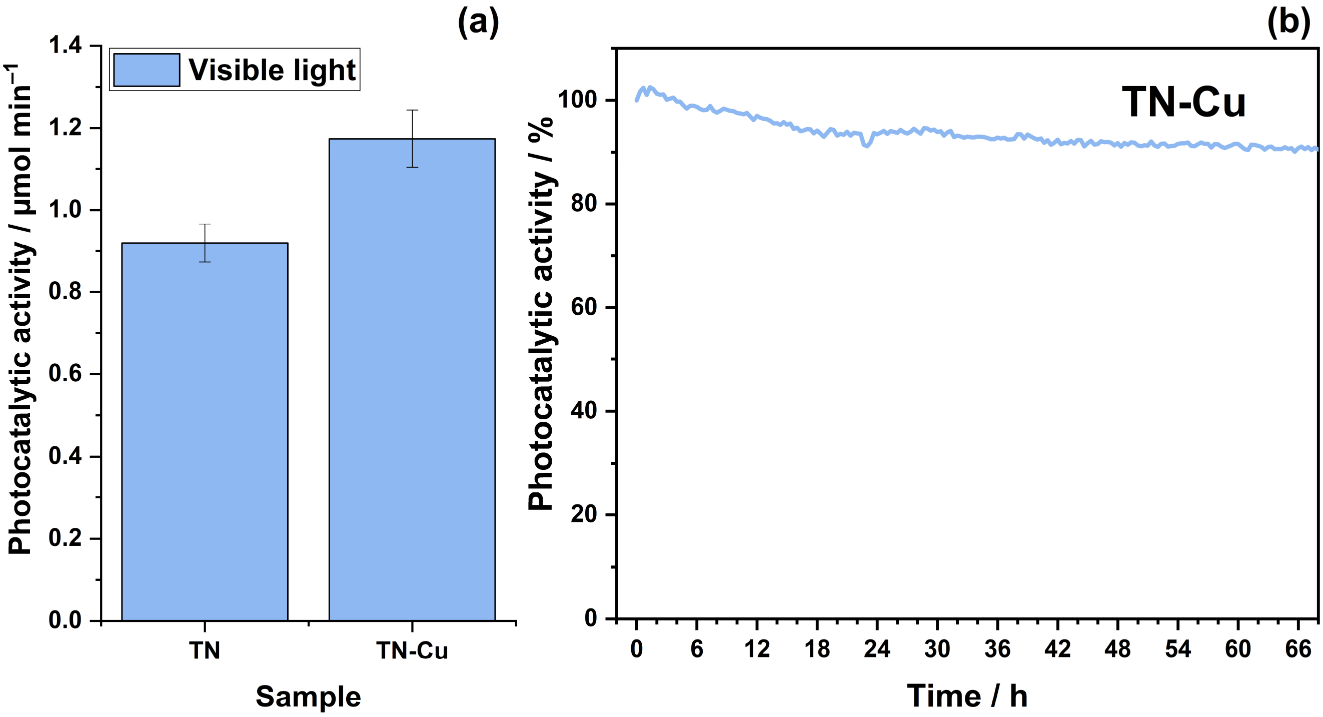

Figure S2. (a) Comparison of initial photocatalytic activity of TiO_2-N (**TN**) powder and Cu-modified TiO_2-N (**TN-Cu**) powder with Cu loading of 1 wt.% in the test reaction of acetone vapor oxidation under visible light; (b) stability of **TN-Cu** photocatalyst in the oxidation of acetone vapor under long-term irradiation.

3. Durability of photoactive PF-TN-Cu fabric after repeated washing

The stability of photoactive coating was studied in several laundry cycles in a washing machine by measuring its photocatalytic activity in the test reaction of acetone vapor oxidation under UVA light after each washing cycle. Briefly, a piece of photoactive **PF-TN-Cu** material was placed in a washing machine (Indesit, Belarus) and washed according to a standard 30-min program proposed by the manufacturer for daily washing using a detergent. Washed piece of fabric was dried at 70 °C for 12 h, and its photocatalytic activity was then evaluated according to the technique described in the main text of manuscript. The procedures of washing, drying, and activity measurement were repeated three times more using the same piece of fabric to monitor a change in the activity. Figure S3 shows that the activity of **PF-TN-Cu** material strongly decreases from 0.29 to 0.22 μmol min^{-1} after the first cycle of washing, probably due to washing out the most weakly bonded particles, but then it decreases slightly and keeps more than 60% (0.175 μmol min^{-1}) initial activity. Then, two additional long-term (1.5–2 h) washing procedures were performed to intensify laundry impact. This long-term treatment leads to a decrease in activity down to 0.13 μmol min^{-1} that corresponds to 40% of initial level but it remains at the same level as the number of washing cycles increases. This result indicates the stability of the prepared photoactivity fabric toward washing procedures.

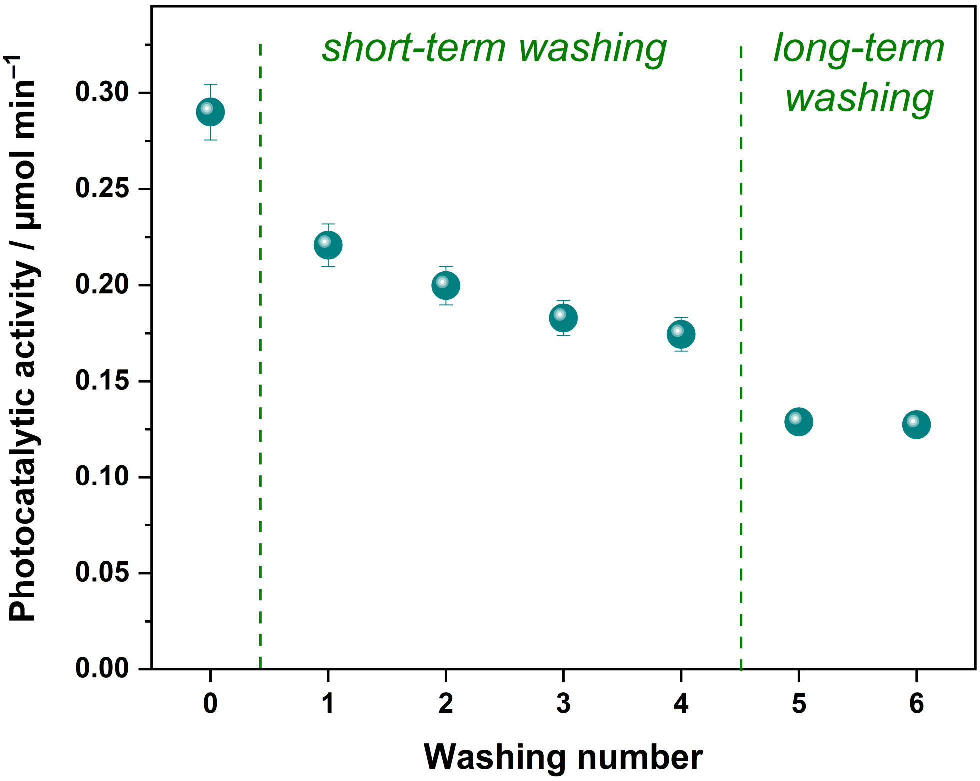

Figure S3. Durability of photoactive fabric after several washing cycles.

4. The obtained threshold cycle value (Cq)

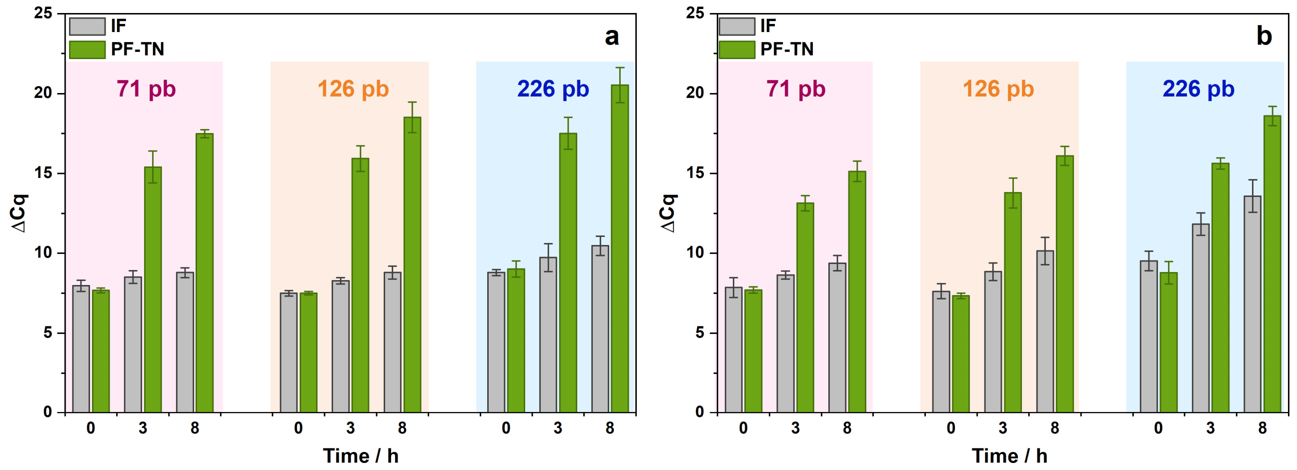

Figure S4. ΔCq of DNA concentration on the surface of initial fabric and PF-TN samples under (a) UV irradiation and (b) visible light.

5. Stability of PA136 bacteriophage

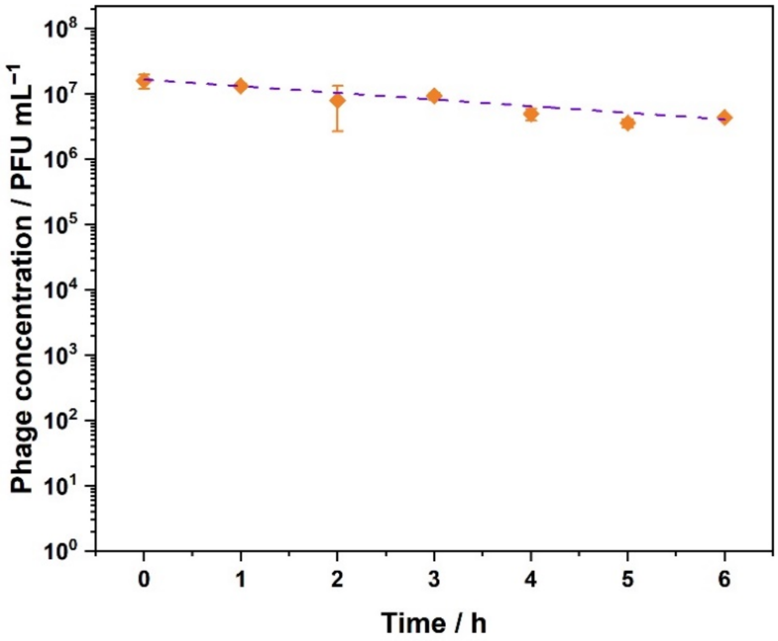

Figure S5. Stability of PA136 bacteriophage in 0.9 wt. % saline solution under UVA radiation in a Petri dish covered with a quartz glass and cooled with a cooling agent.

6. Antiviral activity of photoactive materials

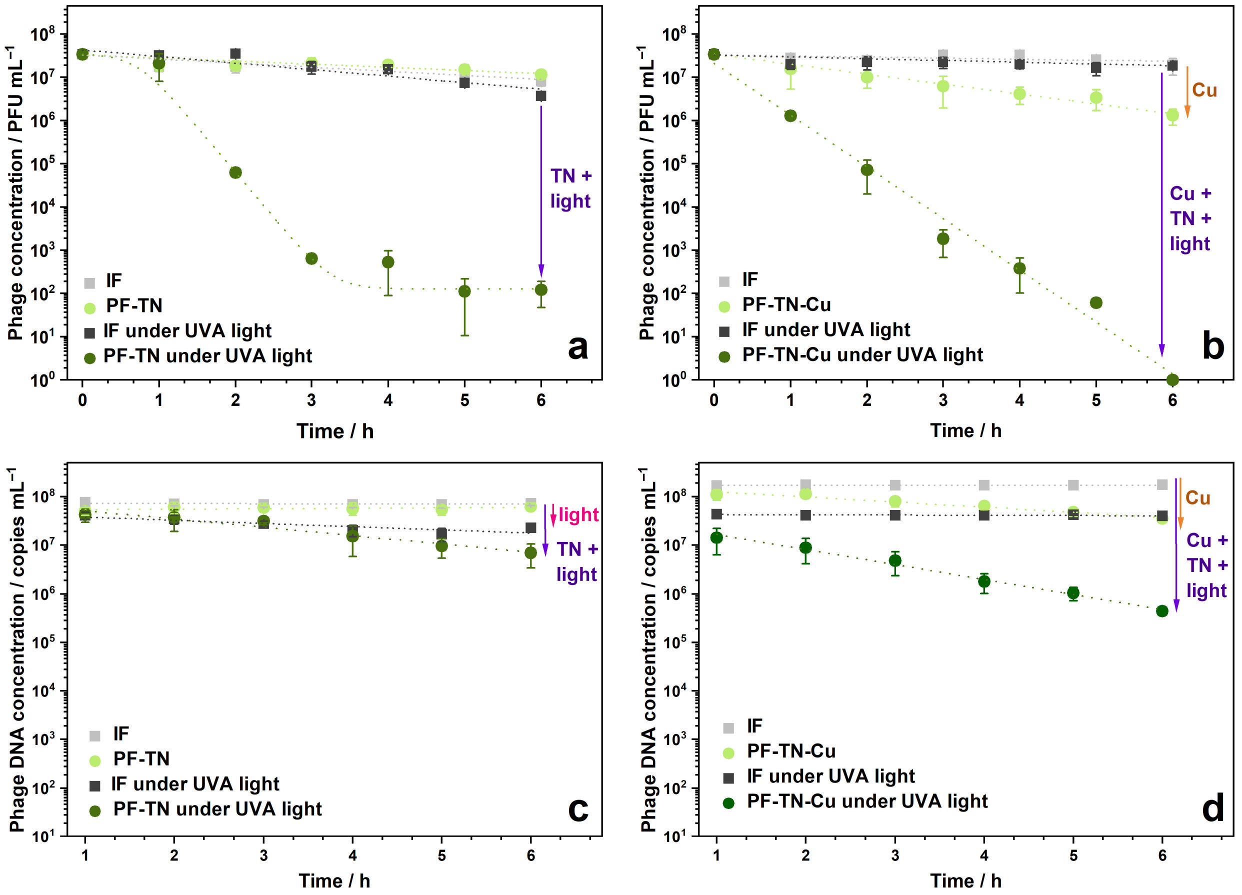

Figure S6. Changes in phage concentration (a) and its DNA (b) on the **PF-TN** and the influence of Cu presence on the phage concentration (c) and its DNA (d) on the **PF-TN-Cu** under UVA irradiation.

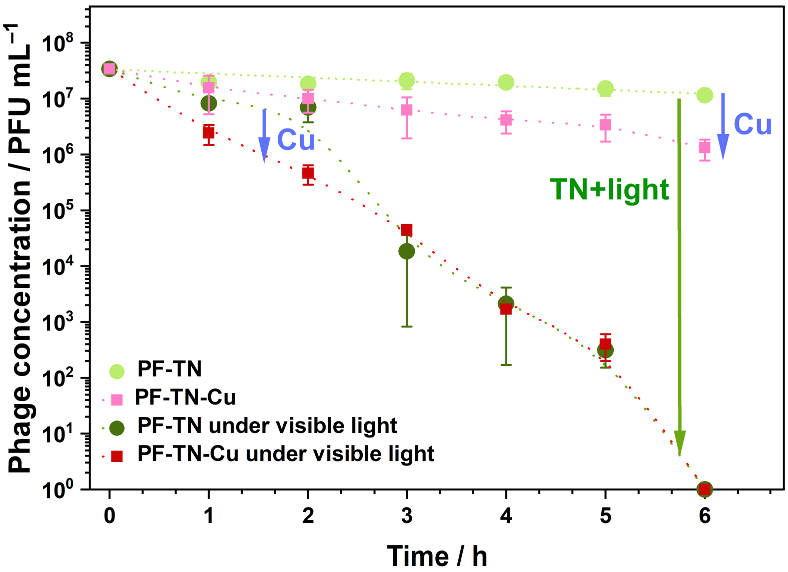

Figure S7. Comparison of kinetic curves for **PF-TN** and **PF-TN-Cu** samples in the dark and under the visible light.

7. Adsorption of *Escherichia coli* on cotton fabrics

The adsorption capacity of cotton-based materials was investigated using the night culture of *E. coli* bacteria (ATCC 25922). Cotton fabric had area density of 350 g·m^{-2}. In addition to initial cotton, the photoactive cotton fabric modified with TiO_2 photocatalyst was used to estimate the effect of titanium dioxide on adsorption. Concentration of bacterial cell was estimated as a number of colony forming units (CFU) in 1 mL of suspension. For these experiments, small pieces with an area of 1 cm^2 were cut from both fabrics and autoclaved at 120 °C for 30 min for the sterilization. A 10-µL aliquot of bacterial suspension (~5·10^6 CFU·mL^{-1}) was dropped on pieces of fabric followed by incubation for 0, 15, 30, or 60 min to analyze the kinetics of adsorption. After that, each piece was transferred into tube with 5 mL of saline. All tubes were vigorously shaken to rinse out the cells from the surface of fabric pieces. The number of living cells was determined by serial dilution with plating on Luria Bertani (LB) agar plates. After incubation at 37 °C overnight, concentration of bacterial cells (CFU·mL^{-1}) was determined according to a standard technique. The number of cells in washout was divided in the total number of cells in initial suspension and multiplied by 100% to evaluate the adsorption capacity of materials. The experiments were repeated at least three times to estimate the error as the standard deviation.

Even short incubation of bacterial suspension on the surface of cotton fabrics led to a strong decrease in the number of cells in washouts (Figure S5). After 15 min of incubation, the materials adsorbed more than 90% of E. coli cells. It is important to note that nanocrystalline TiO_2 used for modification of cotton substantially enhanced the adsorption of bacteria. Further increase in the incubation time led to the complete adsorption of almost all cells on both fabrics. These experiments show a high adsorption capacity of cotton used for functionalization with TiO_2.

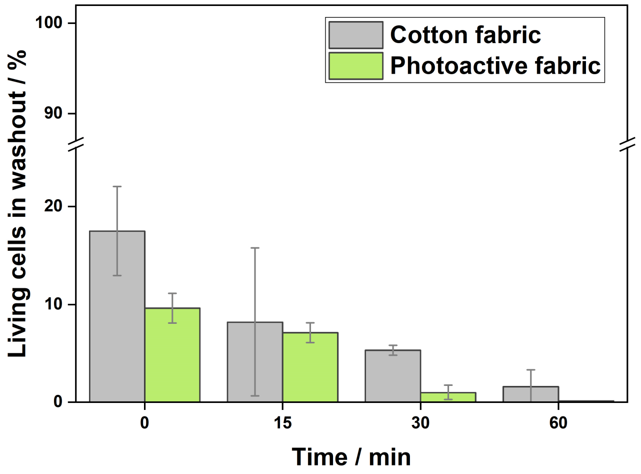

Figure S8. Adsorption kinetics of *E. coli* cells on the surface of cotton and photoactive fabrics.

8. PCR protocol

PCR was performed on a LightCycler® 96 System (Roche Diagnostics, Switzerland) using the following protocol: initial denaturation at 95 °C for 5 min, followed by 35 cycles of denaturation at 95 °C for 10 s, annealing at 60 °C for 10 s, extension at 72 °C for 10 s, and fluorescence acquisition at 81 °C. The final extension step was performed at 72 °C for 5 min. To assess the specificity of the amplified products, a melting curve analysis was carried out by increasing the temperature gradually from 65 °C to 97 °C. The threshold cycle values (Cq) were analyzed using the LightCycler 96 software (version 1.1.0.1320, Roche Diagnostics).

9. Cq and lg of DNA phage concentration dependency

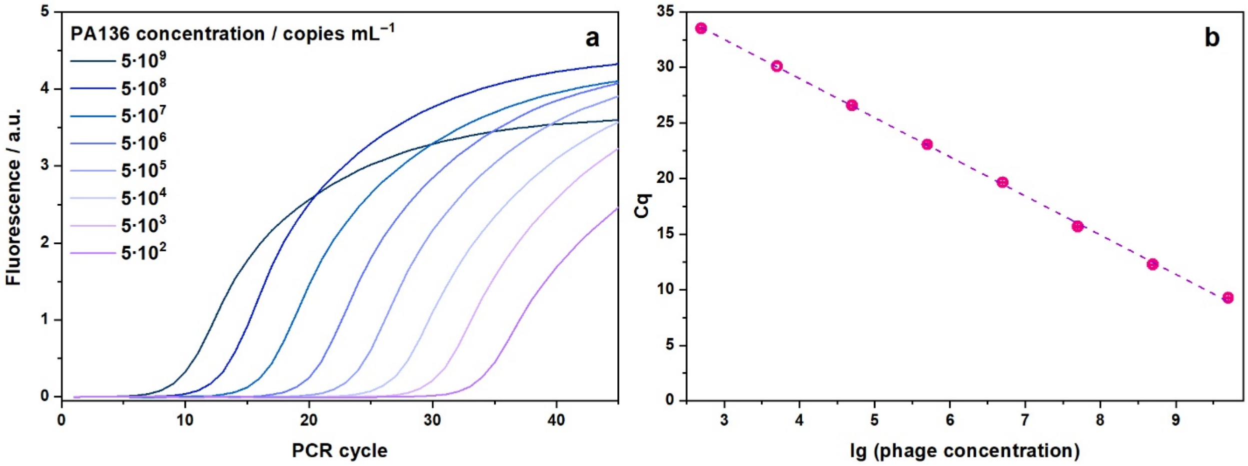

Figure S9. (a) Fluorescence curves of PCR products for sequential tenfold dilutions of bacteriophage PA136 in the range of $5{\cdot}10^2$–$5{\cdot}10^9$ PFU mL^{-1}, (b) standard calibration curve of dependency of Cq on lg of DNA phage concentration obtained by PCR method for sequential tenfold dilutions of bacteriophage PA136 in the range of $5{\cdot}10^2$–$5{\cdot}10^9$ PFU mL^{-1}.
